# Supplementary material for: Beyond opioid prescribing: Evaluation of a substance use disorder curriculum for OBGYN residents
Source: PLoS One. 2022 Sep 15;17(9):e0274563. doi: 10.1371/journal.pone.0274563 (PMC9477269; doi:10.1371/journal.pone.0274563)
Supplement: S1 Appendix — (DOCX) [file pone.0274563.s001.docx]

**S1 Appendix: CREOG objectives addressed by OBGYN residency pilot substance use disorder curriculum**

**Clinical session #1:**

Intrinsic Objectives – Obstetrics
I.F: Describe fetal risk associated with the following: Recreational drugs & Nonprescription drugs

II.E: Counsel patients on breastfeeding

1. Describe the effects of medical and surgical conditions on breastfeeding

2. Understand drug transfer or effect of medication on breastfeeding.

Specialty Objectives

II.C.13: For the following medical conditions, describe the effect of pregnancy and the appropriate screening, diagnosis, and treatment, including indications for consultation or referral: Substance abuse

**Clinical session #2:**

Intrinsic Objectives – General considerations

II. C: Patient Care - Describe behavior patterns suggestive of substance abuse

1. Know appropriate screening tools

2. Initiate therapy with consultation as necessary

II.D: Crisis intervention - Recognize the following: Substance [abuse]

1. Initiate management and appropriate counseling or referral

**Clinical session #3:**

Intrinsic objectives - Office Practice

II.E: Focused Areas in Gynecologic Care - Domestic violence and sexual abuse

1. Define abuse (sexual, physical, and psychologic), violence, substance abuse, and psychosocial abuse; obtain a pertinent history and physical examination; and manage, counsel, and make appropriate referral for patients who are victims of domestic violence, sexual abuse, or both.
