# Supplementary material for: Beyond opioid prescribing: Evaluation of a substance use disorder curriculum for OBGYN residents
Source: PLoS One. 2022 Sep 15;17(9):e0274563. doi: 10.1371/journal.pone.0274563 (PMC9477269; doi:10.1371/journal.pone.0274563)
Supplement: S2 Appendix — (DOCX) [file pone.0274563.s002.docx]

**S2 Appendix**

**Interview guide for OBGYN PGY-1s completing the substance use disorder pilot curriculum**

1. Please describe in your own words what were the most useful components of the novel curriculum on substance use disorders (i.e., self-directed readings, supplemental resources on resident blog, didactics with rotation director in clinic, lectures during resident grand rounds, clinical experiences working with patients, self and instructor evaluations) as part of the perinatal addiction clinical rotation in these two categories:
2. to develop your clinical skills and
3. promote self-directed learning and evidence-based practice
4. Please describe in your own words what were the least useful components of the novel curriculum (i.e., self-directed readings, supplemental resources on resident blog, didactics with rotation director in clinic, lectures during resident grand rounds, clinical experiences working with patients, self and instructor evaluations) plan as part of the clinical rotation in these two categories:
5. to develop your clinical skills and
6. promote self-directed learning and evidence-based practice
7. Now we will go through your responses on the Zwisch Scale one by one and I would like you to explain in your own words why you chose these responses for every question on the scale. For example, you selected “Passive Help” for this question, what do you think we can do we can do to help you become an “Expert”.

For example, you selected “Expert” for this question, can you please explain which components of the curriculum plan helped you become an “Expert”.

1. Please describe in your own words how confident do you feel in screening and treating women with substance use disorders after completing this novel curriculum plan included in your rotation.
   1. How confident are you in your ability to be an independent women’s health provider caring for women with substance use disorders after you finish residency?
2. Please explain in your own words how likely are you to continue caring for women with substance use disorders after you finish your residency?
3. Please describe in your own words any feedback or comments you might have about the novel curriculum on substance use disorders in women’s health?
